# Procedure-Related Access Site Pain Multimodal Management following Percutaneous Cardiac Intervention: A Randomized Control Trial

**DOI:** 10.1155/2022/6102793

**Published:** 2022-01-24

**Authors:** Liuda Brogiene, Aiste Urbonaite, Giedre Baksyte, Andrius Macas

**Affiliations:** ^1^Anesthesiology Department, Lithuanian University of Health Sciences, LT-50009, Kaunas, Lithuania; ^2^Cardiology Department, Lithuanian University of Health Sciences, LT-50009, Kaunas, Lithuania

## Abstract

**Methods:**

137 patients who underwent PCI procedure via radial artery were randomly assigned (1 : 1) to the control (CG, *n* = 68) and intervention (IG, *n* = 65) groups. IG received MPM (paracetamol, ibuprofen, and the arm physiotherapy), CG received pain medication “as needed.” Outcomes were assessed immediately after, 2, 12, 24, and 48 h, 1 week, and 1 and 3 months after PCI. The primary outcome was A-S pain prevalence and pain intensity numeric rating scale (NRS) 0–10.

**Results:**

Results showed that A-S pain prevalence during the 3-month follow-up period was decreasing. Statistically significant difference between the groups (CG versus IG) was after 24 h (41.2% versus 18.5, *p*=0.005), 48 h (30.9% versus 1.5%, *p* ≤ 0.001), 1 week (25% versus 10.8%, *p*=0.042), 1 month (23.5% versus 7.7%, *p*=0.017) after the procedure. The mean of the highest pain intensity was after 2 h (IG-2.17 ± 2.07; CG-3.53 ± 2.69) and the lowest 3 months (IG-0.02 ± 0.12; CG-0.09 ± 0.45) after the procedure. A-S pain intensity mean scores were statistically significantly higher in CG during the follow-up period (Wilks' *λ* = 0.84 *F* (7,125) = 3.37, *p*=0.002).

**Conclusion:**

In conclusion, MPM approach can reduce A-S pain prevalence and pain intensity after PCI. More randomized control studies are needed.

## 1. Introduction 

Ischemic heart disease (IHD) is one of the leading causes of sudden death. Percutaneous cardiac intervention (PCI) is a gold standard to treat IHD. PCI can be performed by the transfemoral or transradial (TR) approach. The TR approach is associated with lower complication rate and better early and long-term outcomes [1]. However, complications such as arterial bleeding, hematoma formation, pseudoaneurysm, or limb dysfunction have been reported in the literature [2, 3]. Complications are accompanied by acute access-site (A-S) pain syndrome. It should be noted that the mechanism of pain is not only related to A-S complications. The development of pain after PCI may be due to hemostasis, concomitant illness (e.g., diabetes, polyneuropathy) of the patient, and possible pain catastrophization [4]. Approximately 1 in 20 patients undergoing PCI experience acute procedure-related A-S pain [5]. It is known that poorly managed acute pain can progress to a chronic condition that results in the disability of the patient. Not only is development of chronic pain associated with the use of abundant analgesics, but also chronic pain tends to impair cardiovascular regulation [6]. Development of chronic pain is associated with many factors, but the most important is severe pain intensity for 24 hours after the intervention and the duration of how long the patient was in pain [7–11]. In patients with IHD, pain management can be a real challenge due to the effect of nonsteroidal anti-inflammatory drugs (NSAIDs) on the cardiovascular system. Most of them are associated with high patient mortality [12]. Inadequate opioid use leads to addiction and other complications associated with their use [13]. Attention should be given to appropriate pain management with risk reduction in cardiovascular events and A-S pain development [14]. The best choice is multimodal pain management (MPM), which is the pain management method when pharmacological and nonpharmacological pain management techniques that act on different pain-inducing mechanisms are selected [15]. We hypothesize that the MPM model will reduce the intensity of pain, the occurrence of pain in patients with CHD after a coronary angiography procedure performed through the radial artery.

## 2. Materials and Methods

### 2.1. Trial Design

Patients who were scheduled for PCI procedure at the Cardiology Department of Hospital of Lithuanian University of Health Sciences Kauno klinikos were recruited in the single-centered RCT. The Regional Ethics Committee approved the trial (approval number BE-2-7, 26^th^ February, 2018). The study was registered at the Australian New Zealand Clinical Trials Registry, ID number: ACTRN12618001699257.

### 2.2. Participants

**Figure 1:**
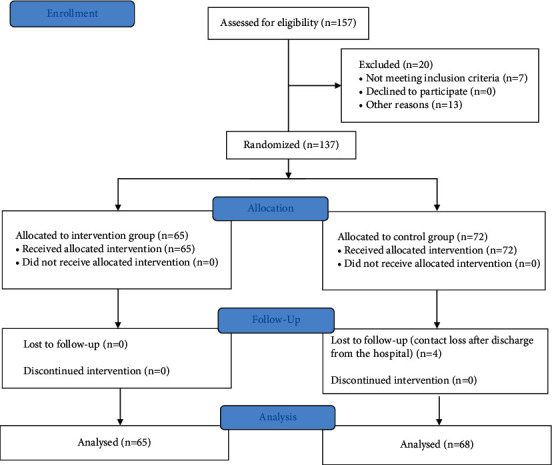
A flow diagram of the study.

All patients provided written informed consent.  Figure 1 shows a flow diagram of the recruitment of the patients' of the study. The first recruitment was on 1st December, 2018 and the last one was on 15^th^ March, 2020. Inclusion criteria were as follows: adults (≥18 years old) with IHD, heart failure classes I-II (Killip/NYHA) who gave written consent, and those who had no allergies or other known contraindications for the use of pain relief medication. Exclusion criteria were as follows: patient refusal to participate in the trial, heart failure classes III-IV (Killip/NYHA), liver, kidney, or other known diseases, or allergies for a medication that will be used, patients who used pain medication before the trial for other pain conditions, A-S pain before the procedure, and pregnancy or breastfeeding.

### 2.3. PCI Procedure

All patients were informed properly about the procedure before the PCI. Access method, sheath size, shape of the guiding catheter, medical therapy, and other materials have been left to the discretion of the operator. When TR method was used, the artery was identified using anatomical landmarks. Local anesthetic (lidocaine 0.5–1 ml 1%) was injected underneath the skin before sheath insertion. After the PCI hemostasis was provided by applying a pressing bandage on the wrist at the puncture site which was started to release 4 hours after the procedure and gradually released until it was safe to remove completely, no other devices were used. All complications were observed clinically and confirmed by the specialist and/or an instrumental investigation.

Hematoma is defined as the presents of a palpable mass greater than 3 cm in diameter measured by a measuring tape and EASY (Early Discharge after Trans radial Stenting of Coronary Arteries Study) hematoma grading scale was used [16]. Hand purpura rush (ecchymosis) discoloration of the skin of the hand without a mass following hemostasis after the PCI procedure. Hand swelling was defined as an arm swelling following hemostasis.

### 2.4. Standard Care and Intervention Treatment

Intervention group (IG) includes pain management with a multimodal approach: regular medication for pain management (2 medications that work synergistically) and physiotherapy. During the PCI procedure, patients get 1 g paracetamol intravenously and ibuprofen 600 mg/day orally during first 24 h after the procedure was given. Patients were trained one-on-one by the physiotherapist to exercise the limb (Supplementary material) (available here). Exercises were started 24 h after the procedure. Patient repeated the exercises 2-3 times a day for one week. There was no heart rate target. If the pain would exacerbate during the exercise, the patient would stop exercising and report to the research team.

The control group (CG) received pain relief in a ‘as needed' regime and it was provided by a cardiologist who was taking care of the patient after the PCI according to the department pain management practice. For the pain management after PCI, ketoprofen intravenously ‘as needed' was used up to a maximal daily dose, if the pain remained weak (tramadol 50 mg orally or intramuscular) or strong opioids (morphine orally or intramuscularly) only in ‘as needed' regime were chosen.

### 2.5. Outcomes

Demographic and baseline data was collected before the PCI procedure. Outcomes were assessed through the procedure and 2, 12, 24, and 48 hours, 1 week, and 1 and 3 months after the procedure. The primary outcome was A-S pain prevalence and pain intensity (NRS, 0–10) during follow-up. Secondary outcomes were A-S complications.

### 2.6. Randomization

The randomized control prospective, parallel-group trial with allocation 1 : 1 was conducted. Randomization (simple randomization protocol) list was created with the Research Randomizer (https://www.randomizer.org/). For the allocation concealment, a sealed envelope was used, which was opened on the day of the procedure by a separate researcher after enrolling the patient.

### 2.7. Sample Size

The sample size was calculated according to the prevalence of acute pain after PCI with an error of 4%, confidence level of 95%, and *α* − 0.05. We estimated that the minimal sample size is at least 39 participants for two queues (total 78) [3, 17].

### 2.8. Statistical Analysis

The data analysis was performed with SPSS statistical software (v. 20.0 IBM). Normally distributed continuous variables were presented as mean ± SD and univariately compared using Student's *t*-test. Categorical data was presented as frequency and percentage and was statistically tested using Chi-square, Fisher's, or Mann–Whitney *U* test where it was appropriate. Multivariate analysis of variance was used to determine whether there were any differences between independent groups on pain intensity means during the follow-up period. Data analysis was as per protocol. All differences were considered statistically significant at a *p* less than 0.05. The risk units have been calculated to have the association strength. For the nominal variables, the contingency coefficient was used, and for ordinal scale, the Kendall tau-c was used.

## 3. Results

The study sample comprised 66% males and 34% females. Patients' mean age in groups was CG 64.1 (±10.5) and IG 64.6 (±12.3) years. Patients in CG had a history of IHD with a mean of 3 years and in IG, 4 years. PCI was performed for stable angina in 60% of cases and the procedure was performed by a senior cardiologist in 80% of cases, and for 40% of cases, the procedure was performed for the first time. Clinical and procedural characteristics of patients are shown in Table 1. The IG got treatment as per protocol and CG “as needed” regime. The “as needed” regime in CG was as follows: during the procedure, 0.9% (*n* = 1) received a strong opioid (morphine, i/v), immediately after 1.5% (*n* = 2) NSAIDs (ketoprofen, i/v) and strong opioids (morphine, i/v); after 2 h, 6.8% (*n* = 9) received NSAIDs (*n* = 7) and strong opioids (*n* = 2); after 12 h, 6% of patients received only NSAIDs (ketoprofen, i/v); after 24 h, no one received pain killers. Also, no side effects of prescribed NSAIDs (in both CG and IG) were observed during the follow-up period. It is important to mention that patients reported nausea and vomiting when strong opiates were given.

**Figure 2:**
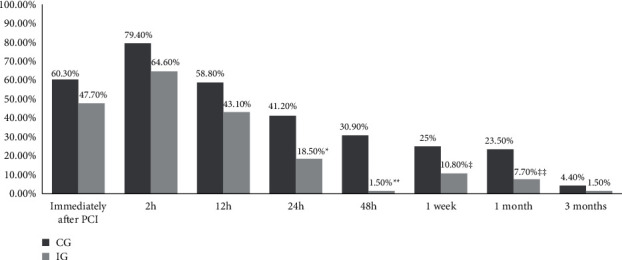
Access site pain prevalence during the follow-up period. PCI: percutaneous cardiac intervention; CG: control group; IG: intervention group; ^*∗*^*p*=0.005; ^∗∗^*p* ≤ 0.001; ^‡^*p*=0.042; ^‡‡^*p*=0.017.

**Figure 3:**
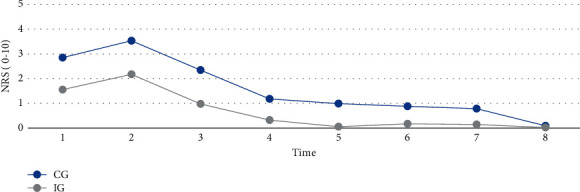
Multivariate comparison showing means of the pain intensity measures between groups (control and intervention) during a 3-month period. The *X*-axis (factor 1) shows time when pain intensity (NRS 0–10) was measured (1, after the procedure; 2, after 2 h; 3, after 12 h; 4, after 24 h; 5, after 48 h; 6, after one week; 7, after one month; 8, after 3 months) and the *y*-axis shows the mean points of the pain intensity. Wilks'*λ* = 0.84 *F* (7,125) = 3.37, *p*=0.002. IG: intervention group; CG: control group; NRS: numeric rating scale.

A-S pain prevalence is shown in Figure 2. A-S prevalence during the 3-month follow-up period was decreasing. The highest point of A-S pain was immediately after (CG-60%, IG-48%), after 2 h (CG-79%, IG-65%), and 12 h (CG-59%, IG-43%) after the procedure. The lowest point during the follow-up period was after 3 months (CG-4.4%, IG-1.5%). The statistically significant difference between the groups was after 24 h, after 48 h, after 1 week, and 1 month after the procedure. The A-S pain intensity mean scores were statistically significantly higher in CG during the follow-up period (Wilks'*λ* = 0.84 *F* (7,125) = 3.37, *p*=0.002). The mean of the pain intensity highest point was after 2 h (IG-2.17 ± 2.07; CG-3.53 ± 2.69) after the procedure and the lowest after 3 months (IG-0.02 ± 0.12; CG-0.09 ± 0.45); see Figure 3.

In order, to see the clinically relevant difference in A-S pain prevalence between IG and CG, the A-S pain intensity was divided into subgroups (NRS): ≤4/10 and >4/10. The intervention group had lower A-S pain prevalence in the subgroup of pain intensity >4/10. Therefore, after 12 h in IG, no one had A-S pain intensity >4/10 (Table 2). During the one-month follow-up period, A-S pain intensity >4/10 prevalence in the CG was 32.4–4.4%. The higher prevalence point of pain intensity >4/10 was after 2 h (32.4%) after the PCI and the lowest after 48 h (4.4%). The A-S pain intensity after 3 months in both groups was ≤4/10.

A-S complications are shown in Table 3. The prevalence of the hand swelling following hemostasis was statistically significantly higher in IG (CG-50% (*n* = 34); IG 72.3% (*n* = 47), *p*=0.008). The swelling disappearance after PCI was shorter (*h*) in IG group (46.7 ± 39.1 versus 29.0 ± 16.6, *p* = 0.045 (*τc*-0.251)). The main A-S complication after the PCI was hematoma: 26% (*n* = 16) in IG and 13% (*n* = 9) in CG (Table 3). Patients in CG who developed A-S complications had A-S pain intensity scores statistically significantly higher in the pain intensity subgroup >4/10 after PCI for 1-month follow-up period (Table 4). The first 2 h after the procedure, IG patients with A-S complications had A-S pain intensity score >4/10 and after 12, 24, 48, 1 week, 1 month, and 3 months, the A-S pain intensity was ≤4/10.

## 4. Discussion

A-S pain after PCI is mentioned in several articles and the problem has been identified in the acute period when the prevalence of severe pain is up to 9.8% [18–22]. This postprocedure pain can be described as a complication associated with the PCI procedure. The mechanism of the A-S pain involves the periprocedural period. It may be induced during the procedure (e.g., vasospasm), in the postprocedure period (bleeding, hematoma formation), and can be hemostasis-related A-S pain, which depends on the kind of hemostasis measures that were used, which may be different in each medical center. According to the pain phenotype, it can be nociceptive or mixed with a component of neuropathic pain [23].

Poorly managed acute pain can progress to a chronic condition that results in patient's disability. Chronic pain is associated with the use of abundant analgesics, and chronic pain also tends to impair cardiovascular regulation [6]. Development of chronic pain is associated with many factors, but the most important is severe pain intensity for 24 hours after the intervention and the duration of how long the patient was in the pain [7–11]. Based on this data, we looked at this problem from an MPM approach.

MPM should be procedure-specific [24]. We chose an MPM model which addresses management of the acute nociceptive or mixed A-S pain syndrome after PCI. This model consists of pharmacological (NSAID combination with paracetamol on the first day) and nonpharmacological (physiotherapy-upper limb exercises 24 h after PCI) measures. For the IG ibuprofen (<1200 mg) was the NSAID of choice. It is known that ibuprofen in combination with paracetamol is the most effective in managing nociceptive pain in the acute postoperative period [25], and it is recommended for low-dose and short-term use in patients with a not complicated cardiovascular disease [26, 27]. The standard treatment in the CG (“as needed” regime) was ketoprofen (NSAID) alone and/or with morphine. Treatment for CG was individually applied by the patient's physician and has not been regulated by the research group. Physicians followed the local hospital guidelines when using strong opioids. Despite the adverse effects of morphine, it has a place in pain relief for patients with acute coronary syndrome (ACS) to prevent further ischemic damages. A. Chen's research group suggests not to change any guidelines or influence physician practice until more randomized control trials will be performed to demonstrate negative outcomes of morphine use in acute pain management for patients with ACS [28].

The findings of Schjerning Olsen et al. indicate that high-risk population after MI is elderly, often treated with NSAIDs, as well as patients, who are at high risk for cardiovascular events and if it is possible, the use of NSAIDs should be avoided in acute pain management period [29]. Cardiovascular risk is variable and depends on the risk of cardiovascular adverse events in patients, choice, and dose of NSAIDs. Therefore, the lowest effective dose of NSAIDs should be used, and treatment should be as short as possible. In 2016, the European Society of Cardiology reported that paracetamol is considered a first-line drug. Naproxen or low dose of ibuprofen (<1200 mg) is preferred if a patient needs NSAIDs and has a medium or high risk of cardiovascular disease [26]. All NSAIDs affect platelets and high doses may increase the risk of bleeding after surgery and can reduce the action of aspirin [30]. It has been known that ibuprofen may interfere with access of aspirin to platelet and may eliminate the protective effect of aspirin [31]. Further research indicates that a degree of inhibition may occur with most NSAIDs and even with some COX-2 inhibitors. The Food and Drug Administration (FDA) stated that ibuprofen should be “given at least 8 hours before or at least 30 minutes after immediate-release aspirin” [32]. However, the age of our study population was about 64 years, no one used the NSAIDs before the procedure for pain issues and the risk of cardiovascular events was very low. We have followed all safety recommendations.

The effect of NSAIDs on renal function in healthy subjects is minimal. Renal adverse reactions occur in 1–5% of all patients receiving NSAIDs. However, patients undergoing PCI with a contrast agent have a higher risk of renal impairment. In addition to changes in blood volume, use of contrast, bleeding, nephrotoxic drugs may also contribute to the development of renal damage after the PCI procedure [33]. However, both acute renal impairment after PCI and chronic kidney disease were associated with poor prognosis. Acute kidney injury (AKI) after PCI was more important than baseline renal function to predict long-term mortality and composite outcomes [34], but none of our patients had renal function impairment before the PCI procedure, and there was no adverse effect noted after the procedure. There is a need for more observational and randomized control studies in AKI after PCI and NSAID use.

The nonpharmacological techniques are another very important part of the MPM approach. A-S complications associated with the procedure (hematoma formation, swelling of the hand due to hemostasis) not only cause A-S pain but also temporarily limit the function of the upper limb. The postprocedure exercise plan, developed with a physiotherapist, reduces the swelling and hasten disappearance of the hematoma. According to our data, swelling after the procedure was 60%, other literature sources indicate the possibility of swelling development as well [35]. It is associated with measures of hemostasis and time of its application after the procedure. Despite the accurate randomization, more patients had swelling in the IG compared to the CG, but the time of disappearance of swelling was better in the IG, which indicates a positive result of the exercise (nonpharmacological approach). It was difficult to estimate the time of hematoma disappearance because the follow-up was via telephone, and it was difficult for patients' self-report regards the hematoma changes.

A-S complications after the procedure can cause pain, discomfort, or even upper limb dysfunction depending on the size of the hematoma formed. In our study, no difference was found between groups in terms of A-S postprocedural complications. The most common complication was the formation of a hematoma at the puncture site, but no significant difference was observed between the groups. The time of applied hemostasis did not differ between the groups as well. Types I-II (according to EASY classification) hematomas were predominated, but patients in the control group experienced higher pain intensity scores (>4/10) throughout the follow-up period compared with IG. The same findings are with other complications. MPM approach gave positive results in pain control in the acute period after the procedure compared with ‘as needed' regime in which even strong opioids were used. Our results showed that pain prevalence and intensity were lower in the IG compared with CG. The statistical and clinical significance is especially noted after 12 hours when the treatment was started, and it reaches the peak after 48 hours and continues up to one month. To find positive results in chronic pain state, a bigger study sample and longer follow-up period with different chronic pain evaluation are needed. We can only hypothesize that good control of the A-S pain intensity and prevalence in acute period can lead to chronic pain prevention.

This study is the first of its kind in postprocedural or procedure-related pain management. Therefore, more RCTs should be performed with other possible pain management models to determine the safest and best MPM model.

The limitations of the study are that it is single-center, not blinded and during the hemostasis period one specific A-S compression method was used. The pain management medication in the CG was individually applied by the patient's physician and was not regulated by the researchers. Also, in this study for pain evaluation, NRS was used which is based on a subjective scale. We used analysis as per protocol and our team was very strict, but despite that, methodology bias cannot be ruled out. We could not follow the patients at home, so we do not know have they performed the exercises as they were taught. We had to trust their positive response over the phone call during follow-up period.

## 5. Conclusion

MPM intervention reduces A-S pain prevalence and A-S pain intensity in the acute period after PCI. Common hemostasis-related complication was arm swelling and a complication related to the postprocedural period was hematoma development. The IG in the subgroup of pain >4/10 demonstrated a reduction of the A-S pain prevalence associated with complications after PCI. The time of swelling disappearance related to hemostasis was shorter in the IG either, but more randomized control studies with a bigger sample are needed.

## Figures and Tables

**Table 1:** Patients demographic and clinical characteristics of control and intervention groups.

Clinical characteristics	Control group *n* = 68	Intervention group *n* = 65	*p*
	*n* (%) or mean ± SD		
Gender (female)	23 (33.8)	22 (33.8)	0.988
Mean of body mass index (kg/m^2^)	28.89 ± 6.05	29.19 ± 4.49	0.241
Mean of age (yrs.)	64.10 ± 10.50	64.63 ± 12.25	0.301
Mean time of IHD (yrs.)	3.28 ± 5.20	4.39 ± 5.58	0.127
Arterial hypertension	61 (89.7)	59 (90.8)	0.836
Diabetes	9 (13.2)	11 (16.9)	0.552
Carpal tunnel syndrome (no use of NSAIDs)	0	1 (1.5)	0.305
Smoking	20 (29.4)	20 (30.8)	0.865
Dyslipidemia	41 (60.3)	38 (58.5)	0.836
Depression	1 (1.5)	1(1.5)	0.974
Rheumatoid arthritis (remission, no use of NSAIDs)	2 (2.9)	2 (3.1)	0.963
Other comorbidities	22 (32.4)	30 (46.2)	0.079
Coronary findings before the procedure			
Unknown	46 (67.6)	38 (58.5)	0.219
Zero-vessel disease	1 (1.5)	2 (3.0)	
Single-vessel disease	7 (10.3)	5 (7.7)	
Double-vessel disease	4 (5.9)	12 (18.5)	
Multivessel disease	10 (14.7)	8 (12.3)	
Procedure			
Diagnostic	5 (7.4)	3 (4.6)	0.347
Stable angina	46 (67.6)	40 (61.5)	
Unstable angina	11 (16.2)	13 (20.0)	
STEMI	4 (5.9)	2 (3.1)	
NSTEMI	2 (2.9)	7 (10.8)	
Procedure performed by			
Senior cardiologist	55 (80.9)	53 (81.5)	0.923
Senior resident	13 (19.1)	12 (18.5)	
Procedure performed			
First time	44 (64.7)	35 (53.8)	0.221
Second time and more	24 (35.3)	30 (46.2)	
Procedure time (min)	34.57 ± 20.33	34.80 ± 20.74	0.826
Coronary findings after the procedure			
Zero-vessel disease	22 (32.8)	24 (37.5)	0.567
Single-vessel disease	12 (17.9)	11 (17.2)	
Double-vessel disease	18 (26.9)	11 (17.2)	
Multivessel disease	15 (22.4)	18 (28.1)	
Number of stents implanted			
0	36 (53.7)	41 (65.1)	0.546
1	18 (26.9)	13 (20.6)	
2	9 (13.4)	8 (12.7)	
3	3 (4.5)	1 (1.6)	
4	1 (1.5)	0	
Systolic blood pressure (mmHg)	150.6 ± 27.12	154.35 ± 22.18	0.127
Diastolic blood pressure (mmHg)	79.56 ± 12.71	78.28 ± 9.25	0.978
Heart rate (bpm)	72.51 ± 12.17	72.23 ± 13.75	0.799
Medications			
Nitroglycerin use during PCI	16 (55.2)	22 (73.3)	0.144
Antiplatelet/Anticoagulants (before PCI)	26 (38.2)	33 (50.8)	0.145
Ticagrelor	3 (4.4)	6 (9.2)	0.594
Clopidogrel	5 (7.4)	3 (4.6)	
Aspirin	12 (17.6)	16 (24.6)	
Aspirin and clopidogrel	2 (2.9)	3 (4.6)	
Anticoagulants	4 (5.9)	5 (7.7)	

IHD: ischemic heart disease; PCI: percutaneous cardiac intervention; STEMI: ST-elevation myocardial infarction; NSTEMI: non-ST-elevation myocardial infarction.

**Table 2:** Access-site pain intensity prevalence during the follow-up period between control and intervention groups according to pain intensity subgroups: ≤4/10 and >4/10.

Pain intensity (NRS)	≤4/10		>4/10		*p*
	Control group	Intervention group	Control group	Intervention group	
Follow-up time					
*n* (%)					
After the PCI	51 (75.0)	56 (86.2)	17 (25.0)	9 (13.8)	0.105
2 h	46 (67.6)	58 (89.2)	22 (32.4)	7 (10.8)	0.003
12 h	55 (80.9)	65 (100)	13 (19.1)	0	<0.001
24 h	64 (94.1)	65 (100)	4 (5.9)	0	0.047^*∗*^
48 h	65 (95.6)	65 (100)	3 (4.4)	0	0.087
1 week	62 (91.2)	65 (100)	6 (8.8)	0	0.028
1 month	64 (94.1)	65 (100)	4 (5.9)	0	0.047^∗∗^
3 months	68 (100)	65 (100)	0	0	—

NRS: numeric rating scale; PCI: percutaneous cardiac intervention; C: contingency coefficient, ^*∗*^C-0.170, ^∗∗^C-0.170.

**Table 3:** The access-site complication distribution between groups.

Access-site complications	Control group *n* = 68	Intervention group *n* = 65	*p*
	*n* (%) or mean ± SD		
*Hemostasis-related*			
Hemostasis time (h)	6.5 ± 2.9	7.1 ± 4.2	0.445
Hand swelling following hemostasis	34 (50.0)	47 (72.3)	0.008

*Swelling disappearance (h)*	46.69 ± 39.1	29.02 ± 16.6	0.045^*∗*^
Purpura rash following hemostasis	24 (35.8)	28 (43.1)	0.394
Purpura rush disappearance (h)	78.55 ± 51.8	58.56 ± 33.9	0.204

*During the procedure*			
Vasospasm	3 (4.4)	4 (6.2)	0.736

*After the procedure*			
Arterial bleeding	3 (4.4)	2 (3.1)	0.686
Hematoma	9 (13.2)	16 (24.6)	0.093
Pseudoaneurysm	1 (1.5)	0	0.326
Infection	0	0	—
Thrombosis	0	0	—
Arteriovenous fistula	0	0	—

*Hematoma (EASY) classification*			
1	3 (37.5)	8 (57.1)	0.596
2	3 (37.5)	5 (35.7)	
3	2 (25.0)	1 (7.1)	
4	0	0	

EASY: Early Discharge after Transradial Stenting of Coronary Arteries Study—access-site hematoma classification [3]; ^*∗*^*τc*-0.251.

**Table 4:** Distributions of the accesses-site complications after PCI according to pain intensity subgroups (≤4/10 and >4/10) during the follow-up period between intervention and control groups.

Follow-up period	Access-site complications	Control group		*P*	Intervention group		*p*
		≤4/10	>4/10		≤4/10	>4/10	
		% (*n*)			% (*n*)		
Immediately after PCI	Hematoma	9.8 (5)	23.5 (4)	0.148	23.2 (13)	33.3 (3)	0.513
	Arterial bleeding	5.9 (3)	0	0.306	3.6 (2)	0	0.565
	Hand swelling fallowing hemostasis	45.1 (23)	64.7 (11)	0.161	73.2 (41)	67.7 (6)	0.684
	Purpura rash following hemostasis	32.0 (16)	47.1 (8)	0.263	42.9 (24)	44.4 (4)	0.929

2 h	Hematoma	0	40.9 (9)	<0.001	20.7 (12)	57.1 (4)	0.034
	Arterial bleeding	2.2 (1)	9.1 (2)	0.194	3.4 (2)	0	0.618
	Hand swelling fallowing hemostasis	34.8 (16)	81.8 (18)	<0.001	72.4 (42)	71.4 (5)	0.956
	Purpura rash following hemostasis	24.4 (11)	59.1 (13)	0.005	43.1 (25)	42.9 (3)	0.990

12 h	Hematoma	7.3 (4)	38.5 (5)	0.003	24.6 (16)	0	—
	Arterial bleeding	1.8 (1)	15.4 (2)	0.032	3.1 (2)	0	
	Hand swelling fallowing hemostasis	40.0 (22)	92.3 (12)	0.001	72.3 (47)	0	
	Purpura rash following hemostasis	29.6 (16)	61.5 (8)	0.031	43.1 (28)	0	

24 h	Hematoma	7.8 (5)	100 (4)	<0.001	24.7 (16)	0	—
	Arterial bleeding	3.1 (2)	25.0 (1)	0.039	3.1 (2)	0	
	Hand swelling fallowing hemostasis	34.8 (34)	0	0.039	72.3 (47)	0	
	Purpura rash following hemostasis	31.7 (20)	100 (4)	0.006	43.1 (28)	0	

48 h	Hematoma	9.2 (6)	100 (3)	<0.001	24.6 (16)	0	—
	Arterial bleeding	4.6 (3)	0	0.704	3.1 (2)	0	
	Hand swelling fallowing hemostasis	47.7 (31)	100 (3)	0.076	72.3 (47)	0	
	Purpura rash following hemostasis	24.4 (21)	100 (3)	0.018	43.1 (28)	0	

1 week	Hematoma	6.5 (4)	83.3 (5)	<0.001	24.6 (16)	0	—
	Arterial bleeding	4.8 (3)	0	0.582	3.1 (2)	0	
	Hand swelling fallowing hemostasis	45.2 (28)	100 (6)	0.010	72.4 (47)	0	
	Purpura rash following hemostasis	31.1 (19)	83.3 (5)	0.011	43.1 (28)	0	

1 month	Hematoma	9.4 (6)	75.0 (3)	<0.001	24.6 (16)	0	—
	Arterial bleeding	4.7 (3)	0	0.658	3.1 (2)	0	
	Hand swelling fallowing hemostasis	46.8 (30)	100 (4)	0.039	72.3 (47)	0	
	Purpura rash following hemostasis	33.3 (21)	75.0 (3)	0.092	43.1 (28)	0	

NRS: numeric rating scale; PCI: percutaneous cardiac intervention.

## Data Availability

To guarantee the confidentiality and anonymity of the participants, data cannot be made publicly available. The protocol and the data are available from the authors. For more information, the corresponding author can be contacted.

## Supplementary Materials

Supplementary Materials Exercises for the limb are shown.
