# Supplementary material for: Procedure-Related Access Site Pain Multimodal Management following Percutaneous Cardiac Intervention: A Randomized Control Trial
Source: Pain Res Manag. 2022 Jan 24;2022:6102793. doi: 10.1155/2022/6102793 (PMC8803434; doi:10.1155/2022/6102793)
Supplement: Supplementary Materials — Exercises for the limb are shown. [file 6102793.f1.docx]

| 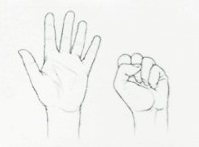 | 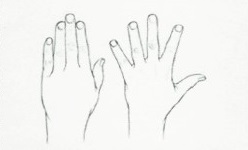 | 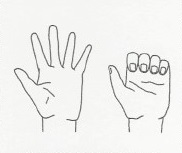 |
| --- | --- | --- |
| Stretch your fingers, then clench them into a fist. | Bring your fingers together, then bring them apart. | Stretch your fingers, then bend them. |
| 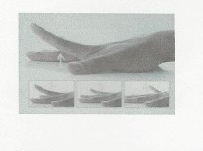 | 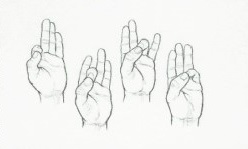 | 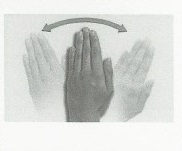 |
| Lean your hand on the table, then lift and lower your fingers one by one. | Attempt to touch the fingertips of your 2^nd^, 3^rd^, 4^th^ and 5^th^ fingers with your thumb. | Tilt your hand leftwards, then rightwards with your wrist joint immobile. |
| 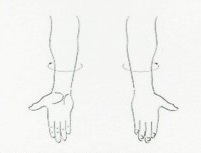 | 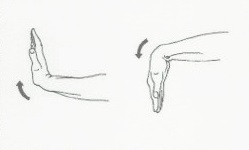  Downward  Upward | 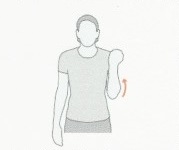 |
| Put both your hands on the table. Rotate both forearms to touch the table with your palm, then with the back of the hand. | Flex your hand upwards and then downwards with your wrist. Do not attempt excessive upward flexion for 7 days after procedure. | Move your arm towards the body (flex the elbow), then move your arm away from the body (extend the elbow). |
| 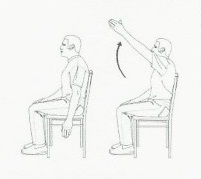 | 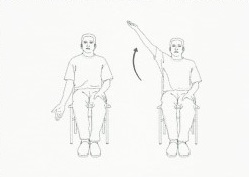 | 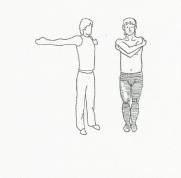 |
| Sit on a chair with a straight back, raise your arm straight up. | Sit on a chair with a straight back, raise your arm straight to the side, then release down. | Extend both your arms to the side, then bring them back (hug yourself). |
| Do the exercises with both arms. Repeat each exercise 10-15 times, 2-3 times per day. | | |
